# Ontario Veterinary College Examinations

**Published:** 1893-01

**Authors:** 


### ONTARIO VETERINARY COLLEGE,
### Toronto, Can.

*Result of the December Examinations—List of Graduates—Notes from the Annual Announcement.*

The December examinations of the Ontario Veterinary College were concluded recently, when the following gentlemen passed a successful examination, and received the diploma of the council of the Agricultural and Arts Association of Ontario :

Charles W. Baker, London, Ont.; Abraham L. Baum, Shelley, Penn., U. S.; Robert S. Beattie, Markham, Ont.; Eugene D. Block, Buffalo, N. Y., U. S.; Elvin L. Button, Durand, Mich., U. S ; William S. Cook, Stouffville, Ont.; James F. Cox, Muscatine, Iowa, U.S.; Henry T. Creagan, Decatur, Mich., U.S.; Thomas E. Early, Aylmer, Ont.; William H. Gaddes, Indian Head, N. W. T.; David Glendinning, Belfountain, Ont.; Charles Wesley Gosnell, Ridgetown, Ont.; William E. McCandless, Capac, Mich., U. S.; James H. McLean, Poplar Hill, Ont.; Clyde L. Sawyer, Kankakee, Ill., U. S.; John W. Smelser, Davenport, Ont.; John B. Stevens, Yale, Mich., U. S.; Thomas Stewart, Boness, Scotland; Charles E. Wright, Grenfell, Assa.

The primary examinations resulted as follows :

*Anatomy*—V. Lathrop, W. J Morgan, F. L. Phelps.

*Materia Medica*—W. R. Hunter, J. M. Klinck, W. J. Morgan.

The new college is a large and spacious building, possessing ample accommodation for the large class of students attending. Its lecture and class rooms are warmed, ventilated and lighted upon the most improved principles to ensure health and comfort. It contains two large lecture rooms, rooms for microscopic and other demonstrations, and every convenience for the thorough teaching of all departments necessary in the equipment of the veterinary surgeon, both as a scientific and practical man. The whole of the new building is so connected with the present college as to give very large and almost perfect accommodation. The establishment forms undoubtedly the finest college building for veterinary purposes in Ameria, and good authorities give it as their opinion that few, even of the great European colleges. can furnish more admirable facilities to their students than are afforded by this college.

A number of the graduates of the college have, during the past year, obtained important appointments in colleges, and as veterinary inspectors in various parts of the United States.

